# Practical Chemistry of Second Year in the Medical Department of the University of Texas

**Published:** 1900-01

**Authors:** 


PRACTICAL CHEMISTRY of Second Year in the Medical Department of the University of Texas. By S. M. Morris, B. S., M. D., Professor of Chemistry and Toxicology, Medical Department University of Texas. Second Edition. Revised and Enlarged. 1898.

This book is companion to one noticed last month, and consists in practical exercises in medical and pharmaceutical chemistry, including systematic examinations of substances for metallic poisons, neutral oils and fats, soaps, etc.; the quantitative and qualitative analysis of the stomach contents, food stuffs, water, drugs, etc. Practical urinalysis is a strong feature of the work. The author has embraced in this little book the outline of a vast amount of practical knowledge, and in his teaching he insists that the student shall understand practical chemistry.

B.

---

E. B. Treat, publisher, is about to issue a book under the title of "Christian Science, an Exposition of Mrs. Eddy's Wonderful Discovery; a Plea for Children and Other Helpless Sick." By William A. Purrington, A. B., LL. M., Lecturer in the University and Bellevue Hospital Medical College (N. Y.) upon Law in Relation to Medical Practice, Etc. This book will be bound in cloth, price $1.00.

